# Integrative multiomics analysis identifies RARRES2 as a regulator of keloid pathogenesis through STAT3/HSPG2 signaling axis

**DOI:** 10.1016/j.isci.2026.115797

**Published:** 2026-04-20

**Authors:** Wenkang Luan, Shujun Fan, Hanyi Jiang, Dongwen Jiang, Jinxiu Yang, Leren He

**Affiliations:** 1 Department of Auricular Reconstruction, Plastic Surgery Hospital, Chinese Academy of Medical Sciences and Peking Union Medical College, Beijing, China; 2 Center for Cleft Lip and Palate Treatment, Plastic Surgery Hospital, Chinese Academy of Medical Sciences and Peking Union Medical College, Beijing, China

**Keywords:** biochemistry, biological database, biological sciences, health sciences, molecular biology

## Abstract

The molecular regulators behind the pathogenesis of keloid have not been fully determined. Through Mendelian randomization and multiomics analyses, we identified retinoic acid receptor responder 2 (RARRES2) as a causal risk factor for keloid. We further found that RARRES2 is mainly enriched in fibroblasts in various human tissues. RARRES2 is highly expressed in keloid tissue and mainly enriched in fibroblasts of keloid. Heparan sulfate proteoglycan 2 (HSPG2) was identified as a potential downstream effector molecule of RARRES2, and p-STAT3 was proven to bind to the promoter region of HSPG2. We demonstrated that RARRES2 promotes the expression of HSPG2 by phosphorylating STAT3. Moreover, we confirmed that RARRES2 exerts the pro-scarring effect through the STAT3/HSPG2 pathway in primary fibroblasts and animal models. Overall, our results demonstrate that RARRES2 is a key regulator of keloid pathogenesis through STAT3-mediated HSPG2 activation, and RARRES2 can serve as a specific molecular target in patients with keloid.

## Highlights

• RARRES2 is identified as a causal risk factor for keloid
• RARRES2 promotes HSPG2 expression via STAT3 phosphorylation
• RARRES2 drives scarring through the STAT3/HSPG2 pathway

## Graphical abstract

**Figure:**
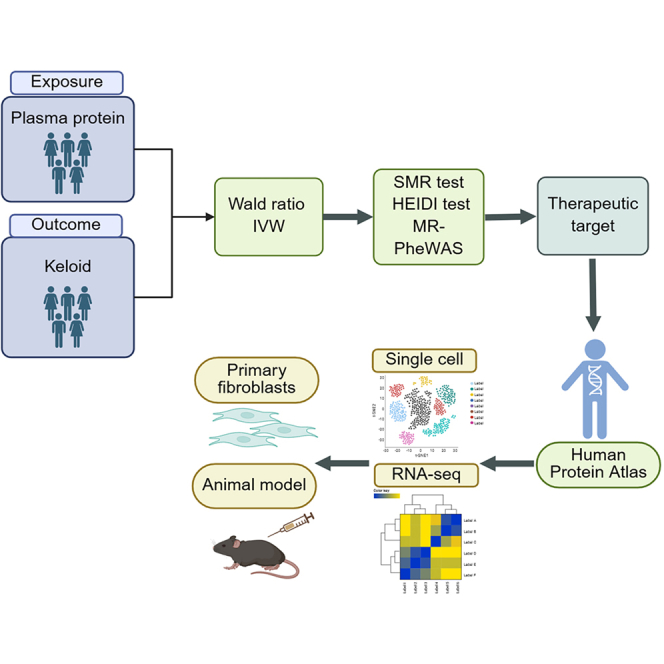

## Introduction

Keloid is a cutaneous fibrosis disease caused by abnormal healing of skin wounds, affecting a significant proportion of the global population.^1^^,^^2^ Besides cosmetic problems, it can cause pain and itching in patients, seriously affecting their life quality.^3^ Keloid cannot disappear spontaneously, and comprehensive treatments such as surgery, laser, and steroid injection are commonly used, but its recurrence rate is still high.^4^^,^^5^ Although keloid is a benign lesion, excessive scar tissue often extends beyond the edge of the initial wound and invades the adjacent normal skin.^6^^,^^7^ Some progress has been made in the study of the pathogenesis of keloid. At present, it is believed that keloid is caused by the abnormal production of extracellular matrix (ECM), leading to excessive collagen deposition, and some regulatory factors of the fibrosis cascade have been found to play an important role.^5^^,^^8^ However, the pathogenesis of keloid is still far from clear.

Mendelian randomization (MR) can use single-nucleotide polymorphisms (SNPs) to infer causal relationships between exposure and disease based on data from genome-wide association studies (GWASs).^9^^,^^10^ MR can reduce the influence of confounding factors^11^ and overcome the influence of reverse causality.^12^ This makes MR a powerful approach for identifying causal circulating protein biomarkers by leveraging large-scale plasma protein quantitative trait loci (pQTL) data.^13^ While GWAS has been used to explore genetic predispositions of keloid,^14^ the systematic application of MR to identify causal protein biomarkers for keloid remains an unmet need. Here, we first used MR to identify plasma proteins that are causally associated with keloid based on pQTL data from three different databases, and identified that retinoic acid receptor responder 2 (RARRES2) is a risk factor for keloid and may serve as a therapeutic target.

RARRES2, also known as Chemerin, is an adipokine that regulates adipogenesis and adipocyte metabolism.^15^^,^^16^^,^^17^ RARRES2 can also play an important role in immune responses by acting as a chemoattractant, recruiting immune cells expressing chemokine-like receptor 1 (CMKLR1).^16^ RARRES2 has been proven to play a role as a tumor suppressor in many cancers,^18^^,^^19^ and is implicated in fibrosis processes, including renal injury-related fibrosis and dental pulp fibrosis.^20^^,^^21^ It has been confirmed that lipid metabolism also plays a key role in the progression of keloid,^22^^,^^23^^,^^24^ but the role of RARRES2 in keloid has not been studied yet. Here, we found that RARRES2 is mainly enriched in fibroblasts in various human tissues, including skin tissue. Fibroblasts play a central role in the formation of keloid; they are continuously activated and secrete ECM in keloid.^25^^,^^26^ We further found that RARRES2 is highly expressed in keloid tissue, particularly in fibroblasts, underscoring its key role in keloid.

Analysis of its downstream molecules revealed that among lipid metabolism-related genes, only heparan sulfate proteoglycan 2 (HSPG2) shows a significant positive correlation with RARRES2 in keloid. HSPG2, the main component of the basal membrane,^27^ has been confirmed to be associated with fibrosis processes in several diseases,^28^^,^^29^ and is closely related to the formation of scar.^30^^,^^31^ In this study, HSPG2 was found to be a hub gene in the protein-protein interaction (PPI) network constructed from 788 key genes of keloid. RARRES2 has been shown to phosphorylate STAT3.^32^^,^^33^ Here, we further demonstrated that RARRES2 exerts its pro-scarring effect by phosphorylating STAT3 to upregulate HSPG2 expression. Overall, RARRES2 may serve as the specific molecular target in patients with keloid.

## Results

### Study design

**Figure 1:**
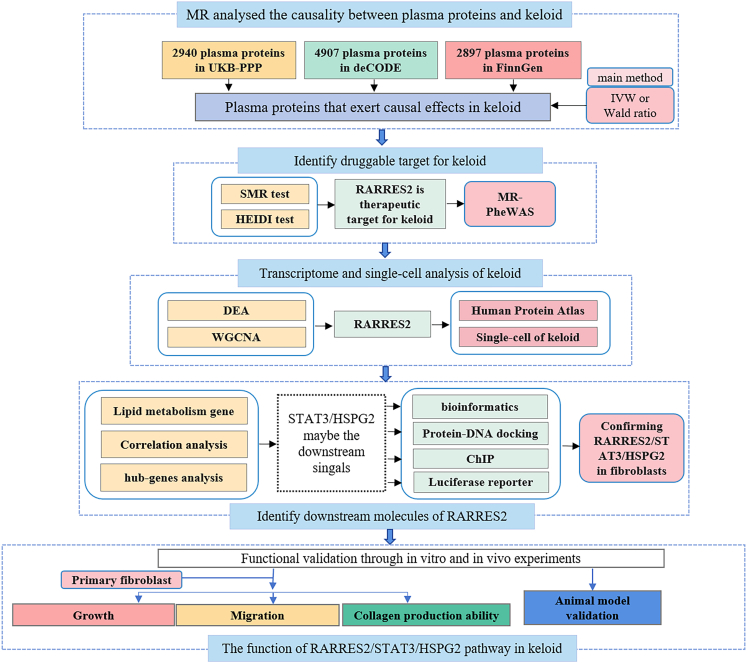
Flowchart of this study design Flowchart of the analytical and experimental methods of this study.

We conducted a two-sample MR analysis to investigate the causal effects of genetically predicted plasma proteins on keloid, using *cis*-pQTLs as instrumental variables across three large-scale proteomic databases. To validate the findings, we performed SMR and HEIDI analyses, MR-PheWAS, and leveraged transcriptomic and single-cell data, followed by experimental validation *in vivo* and *in vitro*. The flowchart of the study design is shown in Figure 1.

### Identifying plasma proteins that exert causal effects in keloid

**Figure 2:**
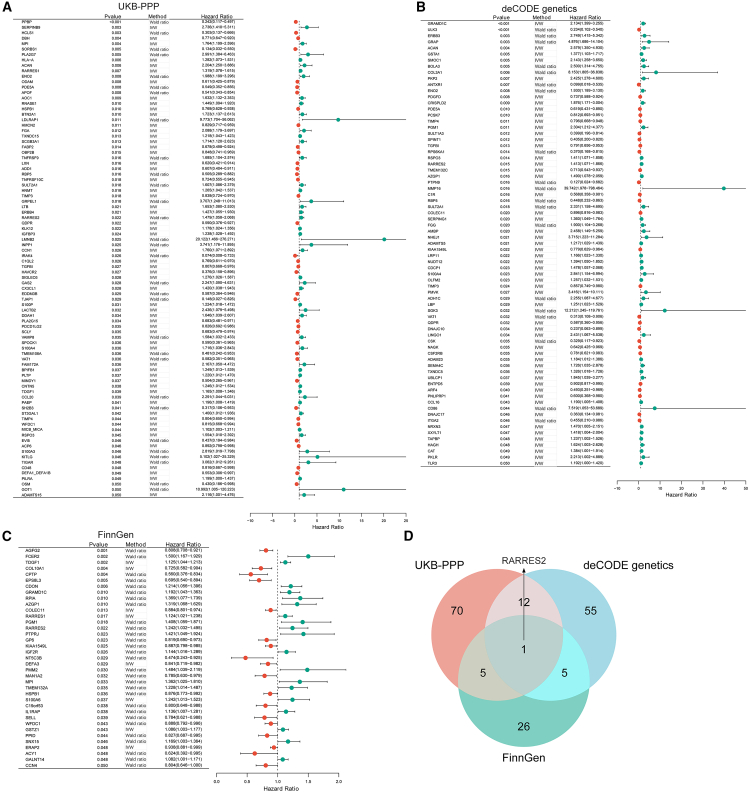
Identifying plasma proteins that exert causal effects in keloid (A) Forest plot showed the Odds Ratio (OR) and 95% Confidence Interval (CI) of 88 plasma proteins that were found to have a causal relationship with keloid through the Wald ratio or IVW methods; the 2940 plasma proteins data came from UKB-PPP. (B) Forest plot shows the OR and 95% CI of 73 plasma proteins that were found to have a causal relationship with keloid; the 4907 plasma proteins data came from deCODE. (C) Forest plot showed the OR and 95% CI of 37 plasma proteins that were found to have a causal relationship with keloid; the 2897 plasma proteins data came from FinnGen. (D) The intersection of meaningful plasma proteins from three different databases.

We first investigated the causal relationship between plasma proteins and keloids. To obtain more reliable and universal results, we included three independent plasma protein GWAS datasets, including 2940 plasma proteins in the UKB-PPP database, 4907 plasma proteins in the deCODE genetics database, and 2897 plasma proteins in the FinnGen database. According to the screening criteria, 5909 SNPs related to 2940 plasma proteins, 5615 SNPs related to 4907 plasma proteins, and 1098 SNPs related to 2897 plasma proteins were included as IVs for MR analysis, respectively, and the detailed information for IVs was shown in Tables S1, S2, and S3. In the UKB-PPP database, we identified 88 plasma proteins that have a causal relationship with keloid through the Wald ratio or IVW methods, including 38 plasma proteins with protective effects and 50 plasma proteins with hazardous effects (Figure 2A). In the deCODE genetics database, we identified 73 plasma proteins, including 28 protective proteins and 45 hazardous proteins (Figure 2B). In the FinnGen database, we identified 18 plasma proteins with protective effects and 19 plasma proteins with hazardous effects, totaling 37 plasma proteins (Figure 2C). We only identified RARRES2 by taking the intersection of the results from the three databases (Figure 2D). RARRES2 has been identified as the risk factor for keloid in all databases (Figures 2A–2C).

### RARRES2 can be used as the druggable target for keloid

**Figure 3:**
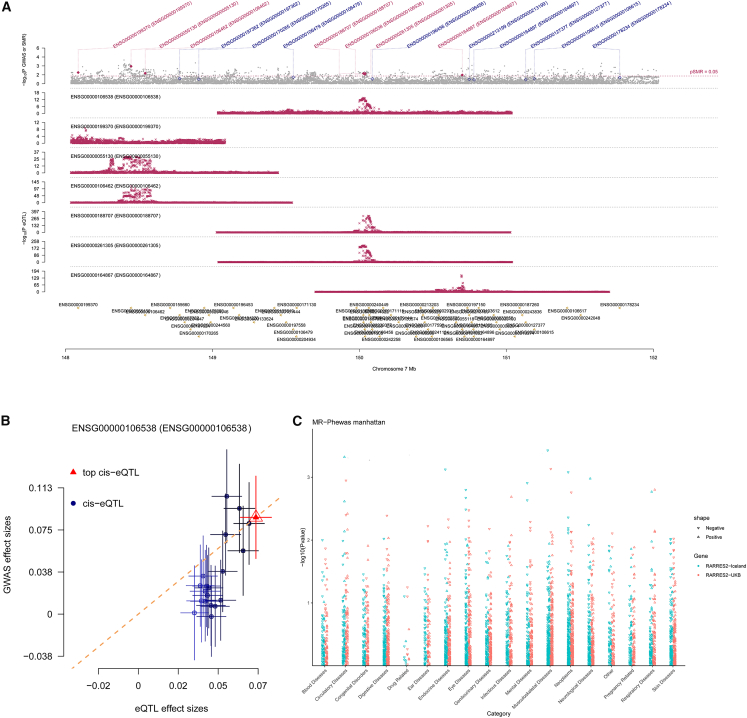
RARRES2 can be used as the druggable target for keloid (A) The SMR locus plots for correlations of RARRES2 in gene expression with keloid. (B) The SMR effects plots for correlations of RARRES2 in gene expression with keloid. (C) Manhattan plot for phenome-wide MR results of RARRES2. Ordinate representation of the *p* value in phenome-wide MR results. A dot represents a disease trait.

We further used SMR and HEIDI to confirm whether RARRES2 is a potential drug target of keloid based on its expression quantitative trait locus (eQTL) data. The eQTL of RARRES2 passed the SMR test (*p* < 0.05) and HEIDI test (*p* > 0.05) (Table S4). The plots of the SMR locus and effect are shown in Figures 3A and 3B. These indicated that RARRES2 can serve as the druggable target for keloid. Moreover, we conducted MR-PheWAS to identify potential side effects of RARRES2 based on the FinnGen R12 database. We did not find a significant relationship between RARRES2 and other phenotypes at the genome-wide level (p < 5e^−05^, -log_10_(P) > 4.301) (Figure 3C and Table S5). These results indicated that if RARRES2 is used as a therapeutic target for keloid, the risk of adverse reactions or unexpected levels of pleiotropy is low.

### RARRES2 may play an important role in keloid tissues

**Figure 4:**
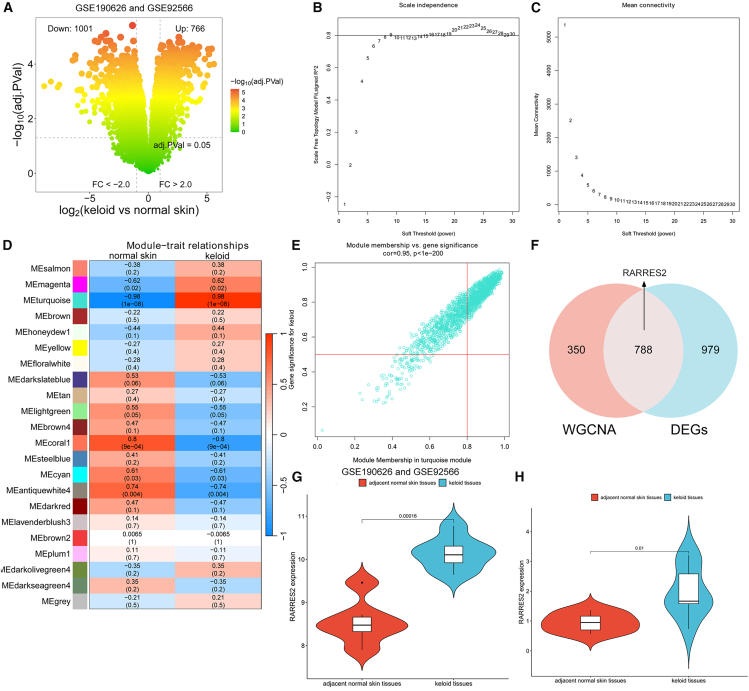
RARRES2 may play an important role in keloid tissues (A) Volcano plot showed all DEGs over 2.0-fold change between 7 keloid tissues and 6 adjacent normal skin tissues (GSE190626 and GSE92566). Upregulated genes were marked in red, and downregulated genes were marked in green. (B) Analysis of the scale-free index for various soft-threshold power. (C) The mean connectivity for various soft-threshold powers. (D) In WGCNA analysis, the heatmap of the correlation between the module eigengenes and keloid. (E) Analysis of gene significance for keloid and module membership in the MEturquoise module. (F) Venn diagram shows the intersection of the DEGs in keloid and the keloid-associated genes in the MEturquoise module. (G) The mRNA expression of RARRES2 in 7 keloid tissues and 6 adjacent normal skin tissues (GSE190626 and GSE92566). (H) The mRNA expression of RARRES2 in 8 keloid tissues and 8 adjacent normal skin tissues.

We next analyzed the genomic profiling in 7 keloid tissues and 6 adjacent normal skin tissues based on public keloid datasets (GSE190626 and GSE92566) to search for differentially expressed genes (DEGs). After removing the batch effects, we found that 1767 DEGs (including 766 upregulated genes and 1001 downregulated genes) showed changes of over 2-fold between keloid tissues and adjacent normal skin tissues (Figure 4A and Table S6). Moreover, WGCNA analysis based on the transcriptome data was used to screen out candidate genes related to keloid. We set the soft threshold to 9 (reaching the platform, R2 = 0.804, slope = −1.590) and constructed the scale-free network (Figures 4B and 4C). We identified 22 modules and the genes they contained after WGCNA analysis (Figure 4D), and the MEturquoise module was found to have the most relevant relationship with keloid (Figures 4D and 4E). Thus, the 1138 hub-genes in the MEturquoise module were considered to be keloid-associated genes (Table S7). We obtained 788 key genes by taking the intersection of keloid-associated genes and DEGs, and RARRES2 is one of these genes (Figure 4F and Table S8). Meanwhile, we found that RARRES2 is upregulated in keloid tissues in both public keloid datasets (GSE190626 and GSE92566) (Figure 4G) and the samples we collected (8 keloid tissues and 8 adjacent normal skin tissues) (Figure 4H). These results indicated that RARRES2 may play an important role in keloid development.

### RARRES2 is mainly enriched in fibroblasts

**Figure 5:**
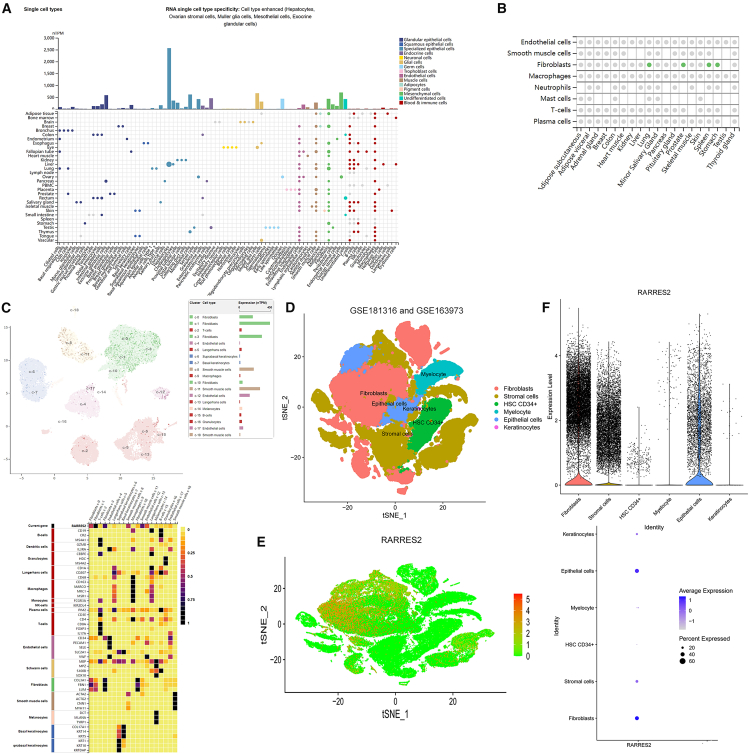
RARRES2 is mainly enriched in fibroblasts (A) The mRNA expression of RARRES2 in different cells of different tissues through Human Protein Atlas (https://www.proteinatlas.org/). (B) The mRNA expression of RARRES2 showed that RARRES2 is mainly enriched in fibroblasts. (C) Single-cell sequencing of skin tissues indicated that RARRES2 is mainly enriched in fibroblasts and smooth muscle cells. (D) The cellular landscape of keloid by analyzing single-cell transcriptomic datasets (GSE181316 and GSE163973). (E) and (F) RARRES2 had the highest enrichment level in fibroblasts among all cell types of keloid tissues.

We identified the expression of RARRES2 in different human tissues through Human Protein Atlas (https://www.proteinatlas.org/), and found that RARRES2 is mainly enriched in fibroblasts of various human tissues (Figure 5A) and has core cell type specificity (Figure 5B). Moreover, in skin tissues, single-cell sequencing indicated that RARRES2 is mainly enriched in fibroblasts and smooth muscle cells (Figure 5C). Similarly, its receptor CMKLR1 was found to be enriched in fibroblasts of multiple tissues, including skin (Figure S1A), and is likewise expressed in vascular smooth muscle cells and fibroblasts within skin (Figures S1B and S1C). We further analyzed the public keloid single-cell transcriptome datasets (GSE181316 and GSE163973) to explore the cellular landscape of keloid tissues. As shown in Figure 5D, the cells in keloid tissue mainly include fibroblasts, stromal cells, HSC CD34^+^, myelocytes, epithelial cells, and keratinocytes. We further found that RARRES2 had the highest enrichment level in fibroblasts among all cell types of keloid tissues (Figures 5E and 5F). Due to the central role of fibroblasts in keloid, these results suggested that RARRES2 may affect keloid formation by influencing fibroblasts.

### HSPG2 has been identified as a downstream molecule of RARRES2

**Figure 6:**
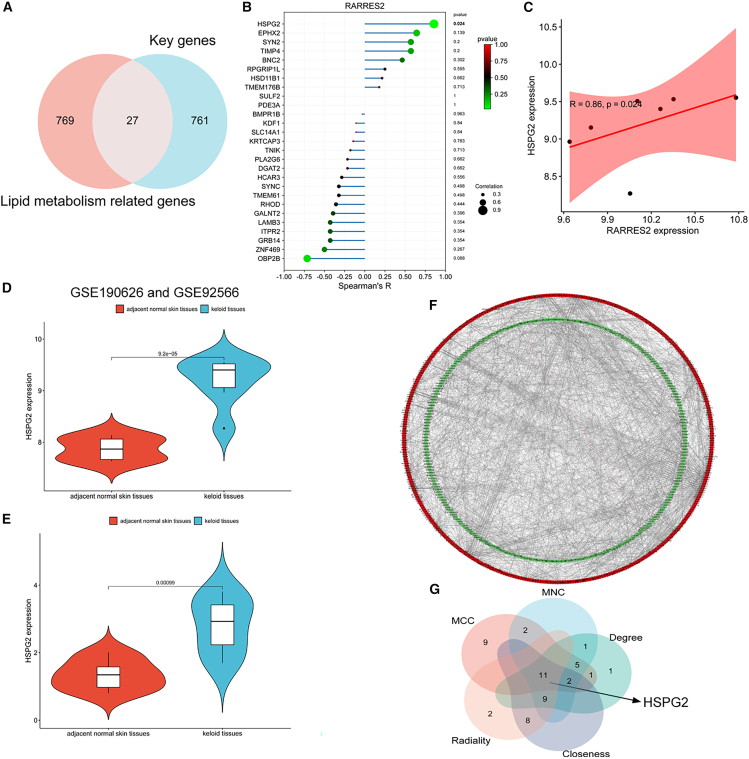
HSPG2 has been identified as the downstream molecule of RARRES2 (A) Venn diagram shows the intersection of the genes related to lipid metabolism and 788 key genes in keloid, resulting in 27 genes. (B) The Spearman’s correlation between RARRES2 expression and all 27 gene expressions in the transcriptome data of keloid (GSE190626 and GSE92566). (C) A significant positive correlation between RARRES2 and HSPG2 in keloid tissues. (D) The mRNA expression of HSPG2 in 7 keloid tissues and 6 adjacent normal skin tissues (GSE190626 and GSE92566). (E) The mRNA expression of HSPG2 in 8 keloid tissues and 8 adjacent normal skin tissues. (F) The PPI network of the 788 key genes was constructed by using the STRING website and the Cytoscape software. (G) CytoHubba plugin in Cytoscape software identified the network hub-genes. Data were expressed as the mean ± SD.

As RARRES2 is a secreted adipokine and lipid metabolism plays an important role in keloid,^22^^,^^23^^,^^24^ we next identified the downstream molecules of RARRES2 in keloid. We first obtained 796 genes related to lipid metabolism from the GeneCards database (https://www.genecards.org/) (Table S9). We took the intersection of these genes with 788 key genes in keloid obtained through differential expression analysis and WGCNA analysis, resulting in 27 genes (Figure 6A and Table S10). We detected the correlation between RARRES2 expression and all 27 gene expressions in the transcriptome data of keloid (GSE190626 and GSE92566), and only found a significant positive correlation between RARRES2 and HSPG2 in keloid tissues (Figures 6B and 6C). We also found that HSPG2 is overexpressed in keloid tissues in both public keloid datasets (Figure 6D) and the samples we collected (Figure 6E). HSPG2 has been confirmed to be closely related to the formation of scar.^30^ To investigate its role in keloid, we constructed the PPI network of 788 key genes by using the STRING (https://cn.string-db.org/) and Cytoscape software to (Figure 6F), and the cytoHubba plugin of Cytoscape was used to score each gene (five methods were used to obtain more reliable results, including MCC, MNC, degree, closeness, and radiality, Table S11). We took the intersection of the top 30 genes with the highest scores from each method and obtained 11 hub-genes of the network, and HSPG2 was among them (Figure 6G and Table S12). These results indicated that HSPG2 is associated with keloid development and may be the downstream molecule of RARRES2.

### RARRES2 promotes the expression of HSPG2 by phosphorylating STAT3 in primary fibroblasts from keloid

**Figure 7:**
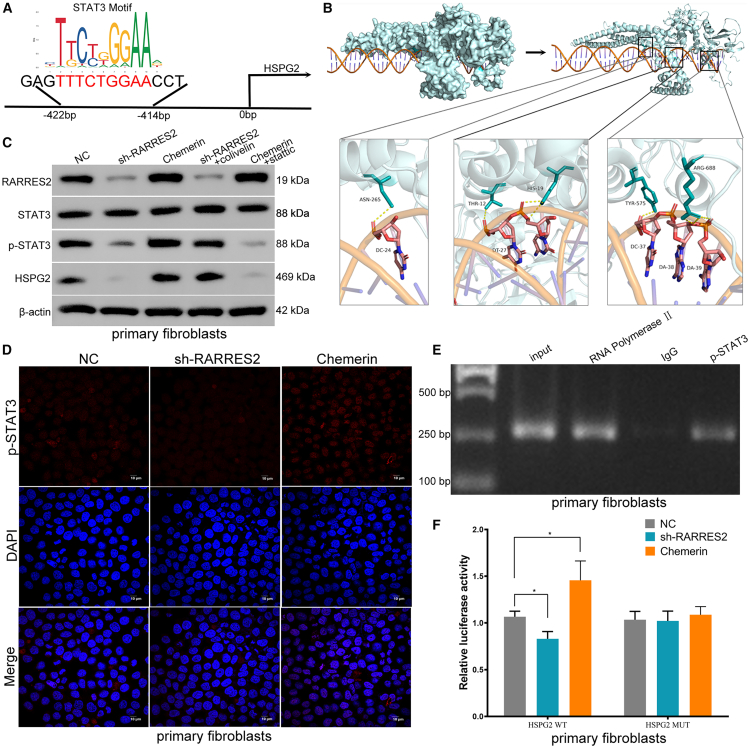
RARRES2 promotes the expression of HSPG2 by phosphorylating STAT3 in primary fibroblasts from keloid (A) There are STAT3 binding sites in the promoter of HSPG2 through the prediction of JASPAR (https://jaspar.elixir.no/). (B) Protein-DNA docking was performed through HDock (http://hdock.phys.hust.edu.cn/), and PyMOL was used to visualize the model and analyze the polar bonds of the link. (C) Western blots identifies the protein level of RARRES2, STAT3, p-STAT3, α-SMA, HSPG2, and β-Catenin in different groups; β-actin was used as a control. (D) IF shows the distribution of p-STAT in and out of the nucleus in different treatment groups. Scale bars, 10 μm. (E) After ChIP, Enriched DNA was amplified by PCR using primers targeting the promoter region of HSPG2. Input DNA and RNA polymerase Ⅱ were positive controls, and IgG was a negative control. (F) Luciferase activity of primary fibroblasts transfected with HSPG2-WT or HSPG2-MUT reporter. The number of biological replicates (n) is 3. Data were expressed as the mean ± SD. Differences were assessed using Student’s *t* test, ∗*p* < 0.05.

We next explored the regulatory mechanism of RARRES2 on HSPG2. It has been confirmed that RARRES2 can phosphorylate STAT3.^32^^,^^33^ Phosphorylated STAT3 can enter the nucleus to activate the transcription of related genes.^34^ We found that there are STAT3 binding sites in the promoter of HSPG2 through the prediction of JASPAR (https://jaspar.elixir.no/) (Figure 7A). In addition, we performed protein-DNA docking through HDock (http://hdock.phys.hust.edu.cn/) based on the protein sequence of STAT3 and the promoter region sequence of HSPG2, and PyMOL was used to visualize the model and analyze the polar bonds of the link (Figure 7B). The molecular docking results showed that STAT3 may bind to the promoter of HSPG2 (Docking Score: −250.63, Confidence Score: 0.8821) (Figure 7B). To further validate the predicted results, primary fibroblasts were extracted from keloid tissues (Figure S2A), and flow cytometry confirmed that primary fibroblasts mainly express fibroblast markers (FSP1, CD90, and vimentin) (Figure S2B). The expression level of p-STAT3 was repressed by the RARRES2 shRNA, and was elevated by Chemerin (also known as RARRES2) in primary fibroblasts (Figure 7C). Immunofluorescence showed that the positive expression of p-STAT3 was increased and transferred more to the nucleus after Chemerin treatment, and these were inhibited by RARRES2 shRNA (Figure 7D). To confirm the binding between p-STAT3 and the promoter region of HSPG2, we conducted ChIP assays in primary fibroblasts. Antibody against p-STAT3 used for immunoprecipitation was able to precipitate the promoter sequences of HSPG2, which can be amplified after a PCR reaction (Figure 7E). These results confirmed that p-STAT3 can bind to the HSPG2 promoter. We next constructed the wild-type and mutant HSPG2 promoter luciferase plasmids containing the STAT3 binding sites, and found that the luciferase activity of the wild-type plasmid was promoted by Chemerin, and suppressed by RARRES2 shRNA in primary fibroblasts (Figure 7F). We further found that the protein expression of HSPG2 was repressed by RARRES2 shRNA and activated by Chemerin (Figure 7C). In addition, colivelin (a p-STAT3 agonist) abolished the effect of RARRES2 shRNA on HSPG2 level, and stattic (a p-STAT3 inhibitor) reversed the promoting effect of Chemerin on HSPG2 expression (Figure 7C). These results indicated that RARRES2 promotes the expression of HSPG2 by phosphorylating STAT3.

### RARRES2 promotes the growth and migration of primary fibroblasts via the STAT3/HSPG2 pathway in keloid

**Figure 8:**
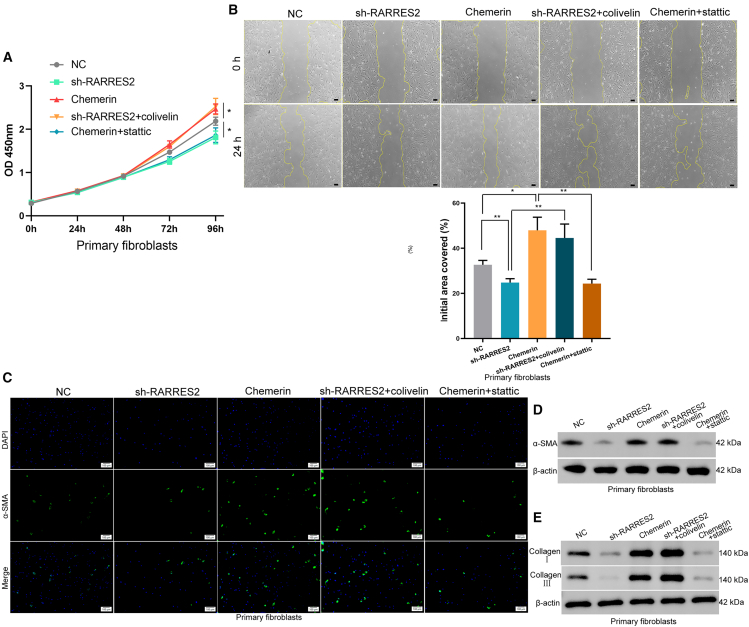
RARRES2 promotes the growth and migration of primary fibroblasts via STAT3/HSPG2 pathway in keloid (A) The proliferative ability of primary fibroblasts was determined by the CCK8 assay in different groups. (B) The migration of primary fibroblasts in different groups was detected by scratch wound assay. *n* = 3. Scale bars, 50 μm. (C) IF assay shows the expression of α-SMA in primary fibroblasts. Scale bars, 100 μm. (D) Western blots identifies the protein level of α-SMA in different groups; β-actin was used as a control. (E) Western blots identifies the protein level of collagen I/III; β-actin was used as a control. Data were expressed as the mean ± SD. Differences were assessed using Student’s *t* test, ∗*p* < 0.05 and ∗∗*p* < 0.01.

We further verify the role of the RARRES2/STAT3/HSPG2 pathway in keloid. CCK-8 assays revealed that RARRES2 shRNA inhibited the proliferation ability of primary fibroblasts (Figure 8A). Scratch wound assays demonstrated that the migrative ability of primary fibroblasts was suppressed by the RARRES2 shRNA (Figure 8B). The immunofluorescence intensity of α-SMA (reflecting the collagen production ability of fibroblasts) was decreased in the RARRES2 knockdown group (Figure 8C). Meanwhile, RARRES2 shRNA suppresses α-SMA protein levels (Figure 8D) and reduces the expression of collagen I and III, along with the collagen I/III ratio (Figure 8E). We also found that Chemerin enhances proliferation, migration, α-SMA expression, and collagen I/III levels, while increasing the collagen I/III ratio in primary fibroblasts (Figures 8A–8E). The role of HSPG2 in promoting the growth and metastasis of primary fibroblasts has also been validated (Figures S3A and S3B). In addition, the ability of Chemerin to promote the proliferation, metastasis, and protein expression of collagen I/III, α-SMA and HSPG2 in fibroblasts was abolished by Statistic (a p-STAT3 inhibitor), while the inhibitory effect of RARRES2 shRNA is reverted by colivelin (a p-STAT3 agonist) (Figures 7C and 8A–8E). These indicated that RARRES2 promotes the growth and migration of primary fibroblasts in keloid via the STAT3/HSPG2 axis.

### RARRES2 promotes the formation of scar through HSPG2 *in vivo*

**Figure 9:**
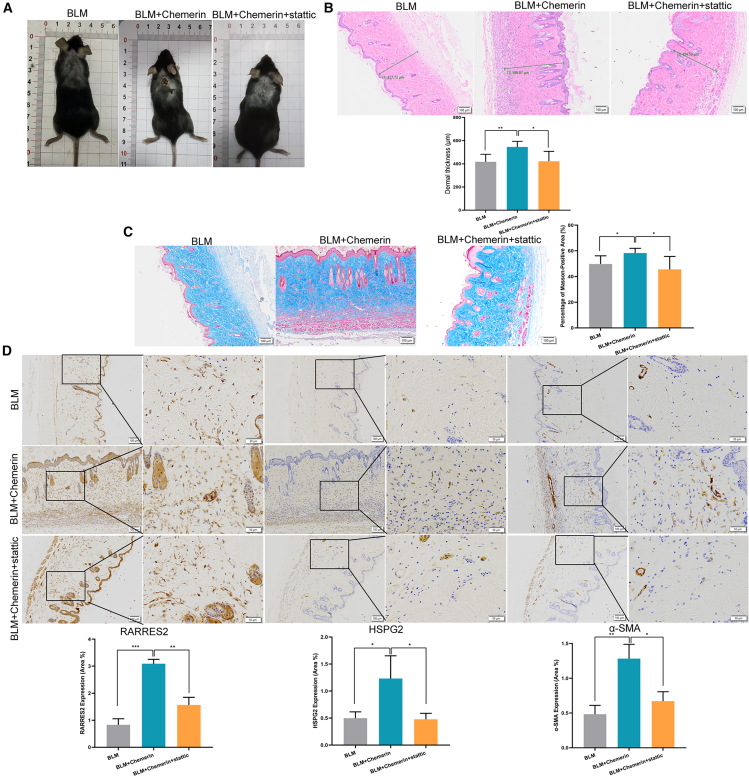
RARRES2 promotes the formation of scar through HSPG2 *in vivo* (A) We used BLM to construct the animal model of scar in 15 mice back skin of 15 mice. Chemerin and statin were used for treatment. We present representative images of different groups. (B) The HE staining of the mouse's back skin. The thickness of the dermis layer has been calculated. *n* = 5. Scale bars, 100 μm. (C) The Masson staining of mice back skin. *n* = 5. Scale bars, 100 μm. (D) The protein level of RARRES2, HSPG2, and α-SMA was detected in the back skin of mice by using IHC. *n* = 3. Scale bars, 100 μm and 50 μm. Data were expressed as the mean ± SD. Differences were assessed using Student’s *t* test, ∗*p* < 0.05 and ∗∗*p* < 0.01.

To confirm the role of RARRES2 in scar formation *in vivo*, we constructed a mouse model with a skin scar by intradermal injection of BLM five times weekly (Figure 9A).^35^ The BLM model enables controllable induction of dermal fibrosis, with reproducible skin thickening and excessive collagen deposition that partially recapitulates fibrotic pathways in keloid. We injected Chemerin and static intradermally into some mice to observe the differences in scar formation (Figure 9A). HE staining showed that Chemerin significantly enhanced the effect of BLM induced increase in dermal thickness (Figure 9B). Masson staining showed that Chemerin also promoted collagen deposition (Figure 9C). Meanwhile, IHC showed that the protein level of RARRES2, HSPG2, and α-SMA was overexpressed in the Chemerin treatment group (Figure 9D). Moreover, static significantly reversed the effects of Chemerin on dermal thickness, collagen deposition, and the expression of HSPG2 and α-SMA (Figures 9A–9D). These results indicate that RARRES2 promotes the formation of scar *in vivo* through HSPG2.

## Discussion

Keloid has become one of the urgent challenges that need to be addressed in clinical practice. Although the histological features of keloid are similar to hypertrophic scar, they have unique growth characteristics, manifested as scar tissue invading adjacent normal skin beyond the edge of the wound, and usually do not spontaneously disappear.^1^^,^^6^ In recent years, many scholars have devoted themselves to studying the etiology and pathogenesis of keloids and have made some progress. It has been confirmed that fibroblasts play a crucial role in the formation of keloid, often in a continuously activated state and secreting excessive ECM.^25^^,^^26^^,^^36^ The process of epithelial-mesenchymal transition (EMT) and the dysregulation of the immune microenvironment are also related to keloid.^37^^,^^38^^,^^39^ However, the molecular driving factors behind the formation of keloids have not yet been determined.

Plasma proteomics was employed to discover systemic biomarkers and druggable targets for keloid management. The bias caused by individual differences profoundly affects the validity of disease research, while MR can provide causal evidence close to randomized controlled trials. MR can avoid bias caused by confounding factors as the separation of genetic alleles follows strict randomization principles when DNA is passed from parents to offspring^11^; MR can minimize reverse causality as the irreversibility of genetics ensures that the association between alleles and diseases remains unchanged.^12^ Here, we employed MR to assess the causal relationship between plasma proteins and keloid. For robustness, we integrated pQTL data from three independent sources: UKB-PPP, deCODE genetics, and the FinnGen database. Integrating results across these databases identified RARRES2 as a risk factor for keloid, highlighting its potential as a therapeutic target.

RARRES2 is a secreted adipokine that regulates lipid metabolism. It can also act as a chemotactic agent by recruiting immune cells to participate in immune responses,^16^ and has been shown to act as a tumor suppressor in many malignant tumors, including adrenocortical carcinoma, breast cancer, glioblastoma, and so on.^15^^,^^16^^,^^40^ Retinoid signaling has been implicated in keloid pathogenesis: AKR1B10 is up-regulated in keloid epidermis and induces an abnormal RA pathway with reduced RA signaling activity, which in turn promotes a profibrotic profile with increased TGF-β1/β2 and collagen I/III expression in fibroblasts.^41^ Recent bulk and single-cell transcriptomic work suggests that retinoic acid can alleviate keloids by inhibiting RBP5-driven pro-inflammatory fibroblasts and reducing collagen secretion.^42^ These data support a broader role of retinoid signaling in keloid biology and provide a conceptual link to RARRES2. However, the role of RARRES2 in keloid has not been studied yet. Here, transcriptomic analyses indicated a key role of RARRES2 in keloid. Its expression was upregulated in keloid tissue and found to be predominantly enriched in fibroblasts, including within keloid single-cell datasets. Given the crucial role of fibroblasts in keloid formation, these results suggest that RARRES2 may affect keloid formation by influencing the function of fibroblasts.

The change of lipid metabolism has been considered a contributor to the pathogenesis of keloid in recent years.^24^ RARRES2 occupies a pivotal position in the core regulation of lipid metabolism. Here, we further identified downstream effector molecules of RARRES2 in keloid. Among lipid metabolism-related genes, HSPG2 is the only gene that was found to have a positive correlation with RARRES2 in keloid tissues. HSPG2 is the core component of the basal membrane and ECM,^27^ and has been shown to be involved in several fibrotic diseases, such as liver fibrosis and systemic sclerosis.^28^^,^^29^^,^^43^ In addition, HSPG2 also plays an important role in scar formation.^30^ In this paper, we validated the high expression level of HSPG2 in keloid tissue and its role in promoting the growth and metastasis of primary fibroblasts. RARRES2 has been shown to activate the JAK2-STAT3 pathway through binding to its receptor CMKLR1.^32^^,^^33^ Moreover, we further found that RARRES2 can phosphorylate STAT3 in primary fibroblasts. Phosphorylated STAT3 can enter the nucleus to activate the transcription of related genes.^34^ We confirmed the binding of p-STAT3 to the HSPG2 promoter and demonstrated that RARRES2 up-regulates HSPG2 via STAT3 phosphorylation in primary fibroblasts. Through this STAT3/HSPG2 pathway, RARRES2 enhances fibroblast growth, migration, collagen I/III ratio, and collagen production, with its pro-scarring effect further validated in animal models. The pathogenesis of keloid involves multifactorial interactions among genetic susceptibility, mechanical stress, and inflammatory pathways. Key pathways such as TGF-β and WNT5A have been validated as drivers of keloid.^35^^,^^44^ Future studies will dissect crosstalk between RARRES2/HSPG2 and intersecting factors.

We integrated plasma proteomics with tissue validation and primary cell experiments to ensure robust identification of biomarkers and therapeutic targets of keloid with both systemic relevance and local pathological accuracy. In view of the high incidence and harmfulness of keloid, our findings have potential clinical significance.

In conclusion, our integrative study combining multi-omics analysis, MR, and experimental validation identifies RARRES2 as a causal risk factor driving keloid pathogenesis through STAT3-mediated HSPG2 activation. These findings establish RARRES2 as a central molecular regulator in keloid formation while delineating its downstream regulatory mechanism. RARRES2 can serve as a specific molecular target in patients with keloid and as a precision medicine strategy for keloid management.

### Limitations of the study

First, we primarily examine the role of biomarkers in fibroblast phenotype modulation, while the contribution of mechanical stress was not explored in depth. Second, although the BLM model can simulate the fibrotic mechanisms of keloid, such as collagen deposition and myofibroblast activation, it cannot fully replicate its specific invasive growth pattern, tissue alignment, and long-term persistence. Third, while our experimental validation was conducted in an East Asian cohort, the large-scale MR analysis primarily utilized data from European populations. This highlights the need for future validation across diverse ethnic groups to confirm the generalizability of the RARRES2-STAT3-HSPG2 axis.

## Resource availability

### Lead contact

Further information and requests for resources and reagents should be directed to and will be fulfilled by the Lead Contact, Leren He (heleren@psh.pumc.edu.cn).

### Materials availability

This study did not generate new unique reagents.

### Data and code availability

Data: The publicly available data used in this study are available in the GEO database under accession codes GSE190626, GSE92566, GSE181316, and GSE163973. Summary-level GWAS data for plasma proteins were obtained from the UKB-PPP, deCODE genetics, and FinnGen database. Keloid GWAS data were sourced from the IEU OpenGWAS project. Detailed sources and identifiers are listed in the STAR Methods section.
Code: This paper does not report original code.
Any additional information required to reanalyze the data is available from the lead contact upon request.

## Acknowledgments

This project was supported by the Fundamental Research Funds for Central Universities at 10.13039/501100011176Peking Union Medical College (3332024147) and Medical and Health Technology Innovation Project of the 10.13039/501100005150Chinese Academy of Medical Sciences (2021-I2M-1-001).

## Author contributions

W.K.L. and L.R.H. conceived and designed the experiments; W.K.L., S.J.F., and D.W.J. collected the public dataset and conducted bioinformatics analysis; W.K.L., S.J.F., H.Y.J., and J.X.Y. performed the experiments; J.X.Y. and H.Y.J. collected samples; W.K.L. and L.R.H. analyzed and interpreted the data. LRH provided the technical support. W.K.L. and L.R.H. drafted the manuscript. L.R.H. critically revised the manuscript for important intellectual content.

## Declaration of interests

The authors declare no competing interests.

## STAR★Methods

### Key resources table

**Table:** 

REAGENT or RESOURCE	SOURCE	IDENTIFIER
**Antibodies**		

Anti-CD90	Biolegend	Cat#389803
Anti-Vimentin	MedChemExpress	Cat#HY-P80371
Anti-FSP1(S100A4)	Proteintech	Cat#16105-1-AP
Goat anti-rabbit IgG H&L ALEXA Fluor@488	Abcam	Cat#AB150077
Anti-RARRES2	Proteintech	Cat#10216-1-AP
Anti-STAT3	Abcam	Cat#ab68153
Anti-p-STAT3	Abcam	Cat#ab267373
Anti-HSPG2	Abcam	Cat#ab318284
Anti-α-SMA	Affinity Biosciences	Cat#AF1032
Anti-Collagen I	Proteintech	Cat#14695-1-AP
Anti-Collagen III	Proteintech	Cat#22734-1-AP
HRP-conjugated secondary antibody	ABclonal	Cat#AS014
Anti-β-actin	Proteintech	Cat#66009-1-Ig
HRP-conjugated goat anti-rabbit IgG polymer	ZSGB-BIO	Cat#PV-9001

**Bacterial and virus strains**		

RARRES2 shRNA lentiviruses	GenePharma	NA

**Biological samples**		

Isolated human primary fibroblasts	This paper	NA

**Chemicals, peptides, and recombinant proteins**		

Chemerin	MedChemExpress	Cat#HY-P70099
Colivelin	MedChemExpress	Cat#HY-P1061
Stattic	MedChemExpress	Cat#HY-13818
Cell counting kit-8	Beyotime	Cat#C0037
Bleomycin	MACKLIN	Cat#9041-93-4

**Critical commercial assays**		

Total RNA Extraction Kit	Solarbio	Cat#R1200
HiScript ® II Q RT SuperMix for qPCR	Vazyme Biotech	Cat#R223
ChIP Assay Kit	Beyotime	Cat#P2078
AceQ qPCR SYBR Green Master Mix	Vazyme Biotech	Cat#Q111
Bicinchoninic acid Protein Assay Kit	KenGen	Cat#KGB2101
The Luciferase Report Assay System	Promega	Cat#E1910
Total Protein Extraction Kit	KenGen	Cat#KGC4901

**Deposited data**		

Transcriptome dataset in keloid and adjacent normal skin tissues	Gene Expression Omnibus	GSE190626
Transcriptome dataset in keloid and adjacent normal skin tissues	Gene Expression Omnibus	GSE92566
The single cell transcriptomic of keloid	Gene Expression Omnibus	GSE181316
The single cell transcriptomic of keloid	Gene Expression Omnibus	GSE163973
GWAS data of 2940 plasma proteins	UK Biobank	NA
GWAS data of 4907 plasma proteins	deCODE genetics database	NA
GWAS data of 2897 plasma proteins	FinnGen database	NA
Keloid GWAS data	IEU OpenGWAS project	NA

**Experimental models: Organisms/strains**		

C57BL/6 mice	Biocytogen	NA
The mice model with skin scar	This paper	NA

**Oligonucleotides**		

Primers used in this study are listed in STAR Methods	General biol	NA

**Recombinant DNA**		

Wild-type HSPG2 promoter-luciferase reporter plasmid	General biol	NA
Mutant HSPG2 promoter-luciferase reporter plasmid	General biol	NA

**Software and algorithms**		

R project	r-project.org	4.3.0
Cytoscape	cytoscape.org	3.10.0
Avogadro	avogadro.cc	1.0
OLYVIA	lifescience.evidentscientific.com.cn	4.1
ImageJ	imagej.org	2.14
GraphPad Prism	graphpad.com	Prism 8
PyMOL	pymol.org	2.x

**Other**		

Quant Gene 9600 Real-time Fluorescence Quantitative PCR Detection System	Bioer	FQD-96C

### Experimental model and study participant details

#### Human tissue samples and public sequencing data

Eight keloid tissues and adjacent normal skin tissues were come from the patients in Plastic Surgery Hospital of Chinese Academy of Medical Sciences and Peking Union Medical College were collected. The patient cohort was of East Asian ancestry, consisting of 5 males and 3 females. Information on patient specific race or ethnicity beyond the broad “East Asian” description, and socioeconomic status was not collected. This study was approved by the Ethics Committee of the Plastic Surgery Hospital of Chinese Academy of Medical Sciences and Peking Union Medical College. The informed consent was obtained from each participant. The public sequencing data were come from the Gene Expression Omnibus (GEO) (https://www.ncbi.nlm.nih.gov/geo/). The published transcriptome dataset in 7 keloid tissues and 6 adjacent normal skin tissues (GSE190626 and GSE92566) was used for further analysis. The single cell transcriptomic of keloid was also obtained from GSE181316 and GSE163973. Human Protein Atlas (https://www.proteinatlas.org/) was used to detect the expression of RARRES2 in human tissues throughout the body. The genes related to lipid metabolism were obtined from the GeneCards database (https://www.genecards.org/).

#### GWAS data

GWAS data of 2940 plasma proteins were come from the study on the relationship between plasma proteomics and human health based on UK Biobank.^45^ The author summarized the plasma proteomic characteristics of 54219 Europeans in UK Biobank and published 2940 plasma protein GWAS datasets, which are deposited on the UKB-PPP website (https://www.synapse.org/Synapse:syn51364943/wiki/622119). GWAS data of 4907 plasma proteins were come from the study on the relationship between the relationship between plasma protein levels and 373 diseases and other traits.^46^ The author summarized the plasma proteomic characteristics of 35559 Icelanders and published 4907 aptamers GWAS datasets, which are deposited on the deCODE genetics database (https://www.decode.com/summarydata/). GWAS data of 2897 plasma proteins were obtained from FinnGen database (https://www.finngen.fi/en).^47^ This database detected the proteomic data of 619 samples based on the Olink platform. Keloid GWAS data was come from IEU OpenGWAS project (https://gwas.mrcieu.ac.uk/), and there are 668 samples from the case group with keloid and 481244 samples from the control group, and the race is European.

#### Mice and construction of animal model

Fifteen 8-12-week-old C57BL/6 mice were purchased from Biocytogen (Beijing, China) and housed under specific pathogen-free conditions with controlled temperature, humidity, and a 12-hour light/dark cycle. To construct the mice model with scar, 100 μg bleomycin (BLM) (MACKLIN, Shanghai, China) was intradermally injected into the back skins of mice, the frequency was five times weekly for 3 weeks. 4 μg/kg Chemerin (MCE, New Jersey, USA) was intradermal injected into the back skins of the mice four times weekly for 3 weeks, and 5 mg/kg stattic (MCE, New Jersey, USA) was intradermal injected into the back skins of the mice two times weekly for 3 weeks. The mice were euthanized and their back skin was taken for subsequent experiments after 3 weeks. This study was approved by the Ethics Committee of the Plastic Surgery Hospital of Chinese Academy of Medical Sciences and Peking Union Medical College.

### Method details

#### Study design description

We performed two-sample MR analysis to assess causal relationships between genetically predicted plasma proteins and keloid, using cis-acting protein quantitative trait loci (cis-pQTLs) as IVs. 2940 plasma proteins in UKB-PPP database, 4907 plasma proteins in deCODE database and 2897 plasma proteins in FinnGen database were considered as the exposures, with keloid serving as the outcome. Subsequently, Summary-data-based MR (SMR) and Heterogeneity in Dependent Instruments (HEIDI) analyses and MR phenome-wide association study (MR-PheWAS) analyses were conducted. The transcriptome sequencing and single-cell transcriptome data of keloid and Human Protein Atlas (https://www.proteinatlas.org/) were used for further identify key pathways in keloid. Finally, *in vivo* and *in vitro* experiments were conducted to validate the result. The flowchart of the study design is shown in Figure 1.

#### Selection of instrumental variables (IVs) for MR

When selecting effective IVs, three core assumptions must be met in principle, including association assumption, independence assumption and exclusive restrictions. The screening criteria are as follows: P <5×10^-8^ for SNPs is the basic criterion, SNPs with linkage disequilibrium (kb=10000, r2>0.001) and SNPs with F<10 (weak instrument strength) were excluded, and cis-regulatory window is 1000 kb.

#### MR causal effect estimation

We excluded SNPs that being palindromic with intermediate allele frequencies when harmonizing outcome and exposure data.^48^ When there are multiple SNPs remaining, 4 MR methods (IVW, Weighted mode, MR-Egger and Weighted median) were used to evaluate the causal relationship between plasma proteins and keloid; when there is single SNP remaining, the Wald ratio was used. In the absence of pleiotropy, IVW is the primary method and other methods are supplementary, as it assumes that instruments can only affect the results through exposure rather than other alternative pathways.^48^^,^^49^ The sensitivity analyses we conducted included heterogeneity test, pleiotropy test, and leave-one-out sensitivity analysis. Specifically, Cochran's Q test in MR-Egger and IVW were used to evaluate heterogeneity, and MR-Egger 's intercept was used to evaluate pleiotropy, and Leave−one−out sensitivity analysis was used to evaluate whether a single SNP affects the overall MR results.

#### SMR and HEIDI test

SMR and HEIDI tests can distinguish between pleiotropic models and linkage models.^50^ As a supplementary method, SMR analysis was used to confirm the causal relationship between keloid and Expression Quantitative Trait Loci (eQTL, it obtained from eqtlGEN, https://www.eqtlgen.org/), and P < 0.05 was defined as significant correlation ^51^. HEIDI test was used to demonstrate that proteins associated with keloid are not caused by genetic linkage, with P > 0.05 indicating that this association is due to shared genetic variations.^51^

#### MR-PheWAS

In order to study the potential side effects of druggable targets, we used plasma proteins as exposure and summary data of 2469 diseases in the FinnGen R12 as outcomes to perform MR-PheWAS. We performed MR analysis with the same parameters via the Wald ratio or IVW method. When p<5e^-05^, causal effects are considered statistically significant. It provides insights into understanding complex genetic features and evaluating the safety and efficacy of druggable targets.

#### Bioinformatics analysis

For Differential Expression Analysis (DEA), we analysed transcriptomic profiling to identify differentially expressed genes (DEGs) in keloid. Sva and limma packages in the R project were used to merge data and remove batch effects, and NormalizeBetweenArrays algorithm, Pheatmap and ggplot2 packages in R software was used. For Weighted Gene Co-expression Network Analysis (WGCNA), we used the WGCNA package and identified modules and genes within the modules that have significant clinical relevance to keloid. For detecting the cellular landscape and transcriptome in keloid tissues, SingleR package in R software was used. The PPI relationships of key genes were detected through STRING website (https://cn.string-db.org/). Based on the PPI relationship, Cytoscape software was used to construct the PPI network and cytoHubba plugin was used to calculate and identify hub-genes of PPI network (five methods were used, including MCC, MNC, Degree, Closeness and Radiality). The Spearman correlation analysis was performed to detect the correlation of gene expression.

#### Bioinformatics prediction and Protein-DNA docking

We obtained the promoter sequence of HSPG2 gene from UCSC (https://genome.ucsc.edu/) and used JASPAR (https://jaspar.elixir.no/) to predict the binding sites of STAT3 and HSPG2 promoters. Avogadro software was used to generate the 3D structure of promoter sequences based on binding sites. We obtained the protein sequence of STAT3 from the UniProt (https://www.uniprot.org/) and perform hydrogenation using PyMOL software. HDock (http://hdock.phys.hust.edu.cn/) was used to conduct protein-DNA docking and Rank1 model was selected for visualization. We visualized the model using PyMOL software and analyzed the polar bonds of the link. Docking Score represents the molecular docking score, and Confidence Score represents the reliability of the model.

#### Extraction of primary fibroblasts, cell culture, biological reagents, oligonucleotides

Keloid tissue was soaked in 75% alcohol for 1 minute and washed with Phosphate Buffered Saline (PBS). We cut the tissue into small pieces with a volume of about 1mm^3^ after removing fat and epidermis. Then, a small amount of Fetal Bovine Serum (FBS, Pricella, Wuhan, China) was added to tissue pieces, and the tissue pieces were inoculated in culture dish and cultured for 24 hours to adhere to the wall. Dulbecco's modified Eagle's medium (DMEM)/High Glucose (Cytiva, USA) with 10% FBS was then used to incubate cells for 3-4 weeks, and primary fibroblasts were grown in DMEM/High Glucose with 10% FBS. Cells were placed in the humidified cell incubator with 5% CO2 at 37°C. Chemerin were purchased from MedChemExpress (New Jersey, USA) and the working concentration in the culture medium is 50 ng/ml, colivelin (a p-STAT3 agonist) were obtained from MCE (New Jersey, USA) and the working concentration is 50 μg/mL, and stattic (a p-STAT3 inhibitor) were obtained from MCE (New Jersey, USA) and the working concentration is 5 μM. The RARRES2 short hairpin RNA (shRNA) lentiviruses were synthesized by GenePharma (Suzhou, China), and the sequence as follows: shRNA1, 5'-GCCCTTCCCAGCTGGAATATT-3'; shRNA2, 5'-GCTTCTACTTCCCTGGACAGT-3'.

#### Flow cytometry

Flow cytometry was used to detect the expression of cell markers in primary fibroblasts. Antibodies against CD90 (direct labelled) was obtained from Biolegend (California, USA). Antibodies against Vimentin (MCE, New Jersey, USA) and FSP1 (Proteintech, Chicago, USA) was used to incubate cells, followed by incubating with secondary antibody (goat anti-rabbit IgG H&L ALEXA Fluor@488, Abcam, Cambridgeshire, UK). The isotype control antibody was obtained from Biolegend (California, USA).

#### Cell proliferation and cell migration assays

Cell counting kit-8 (CCK-8, Beyotime, Shanghai, China) assay was used to evaluate cell proliferation ability. Primary fibroblasts were inoculated into the 96 well plate with the density is 3000 cells/well. We added the CCK8 solution into each well and incubated cell for an additional 2 hours at 0 h, 24 h, 48 h, 72 h, 96 h. The microplate reader was used to measure the absorbance (optical density is 450nm). Scratch wound assay was used to evaluate cell migration ability. The primary fibroblasts were inoculated into the 6-well plates, and the cells were scratched to create a wound space using a 200 μl pipette. At 0 and 24 h after scratching, the wound width was recorded and photographed. ImageJ software was used to evaluate changes in scratch area.

#### Quantitative RT-PCR (qRT-PCR)

Total RNA was extracted by using Total RNA Extraction Kit (Solarbio, Beijing, China). HiScript ® II Q RT SuperMix for qPCR (Vazyme Biotech, Nanjing, China) was used to conduct Reverse transcription (RT) reaction, and AceQ qPCR SYBR Green Master Mix (Vazyme Biotech, Nanjing, China) was used to perform qRT-PCR reaction. Quant Gene 9600 Real-time Fluorescence Quantitative PCR Detection System (Bioer, Hangzhou, China) was used for amplification reactions according to the reaction condition. The primers were synthesized with the following sequences: RARRES2, 5'-CTACAGGTGGCTCTGGAGGAGTTC-3' (forward) and 5'-TCAGCTCTGTCCACACCGATCTC-3' (reverse); HSPG2, 5'-GACGGCTCTTTCCACCTGAG-3' (forward) and 5'-CGACTGACACCCATGCAGAA-3' (reverse); GAPDH, 5'-ACCCAGAAGACTGTGGATGG-3' (forward) and 5'-TTCAGCTCAGGGATGACCTT-3' (reverse). GAPDH was used for normalization. The calculation method of relative expression is 2^–ΔΔCt^.

#### Western blot analysis

Total proteins were extracted from samples using Total Protein Extraction Kit (KenGen BioTech, Nanjing, China) according to the manufacturer's instructions. Protein concentrations were quantified using Bicinchoninic acid Protein Assay Kit (KenGen BioTech, Nanjing, China). Equal amounts of protein were loaded onto the SDS-PAGE gels and separation was performed according to the conditions. Proteins were then transferred to polyvinylidene fluoride membranes (Millipore, Billerica, USA). The membranes were blocked with 5% non-fat milk and incubated overnight at 4°C with antibodies against RARRES2 (Proteintech, USA), STAT3 (Abcam, Cambridgeshire, UK), p-STAT3 (Abcam, Cambridgeshire, UK), HSPG2 (Abcam, Cambridgeshire, UK), α-SMA (Affinity Biosciences, UK),Collagen I (Proteintech, USA), Collagen III (Proteintech, USA). Membranes were incubated with appropriate HRP-conjugated secondary antibody (ABclonal, Wuhan, Chian) for 1 h at room temperature. β-actin was used for normalization (Proteintech, USA).

#### Immunofluorescence (IF)

We fixed the cells with paraformaldehyde and performed cell permeability by using 0.5% TritonX100. We then blocked it with 3% bovine serum albumin and incubated it overnight at 4°C with the first antibodies against α-SMA (Affinity Biosciences, UK) and p-STAT3 (Abcam, Cambridgeshire, UK). Next, we incubated it with a second antibody (ZSGB-BIO, Beijing, China) for 1 h at room temperature, and stained the cell nucleus with DAPI. Finally, images were taken under fluorescence microscopy and confocal microscopy.

#### ChIP

We fixed protein-DNA interactions in primary fibroblasts using formaldehyde. ChIP Assay Kit (Beyotime, Shanghai, China) was used for the ChIP assay. According to the instruction, we sequentially added Glycine Solution, PBS, SDS Lysis Buffer into the cells, and used ultrasound to shear chromatin into fragments of 200-1000bp. We then incubated chromatin fragments with ChIP primary antibodies against p-STAT3 (Abcam, Cambridgeshire, UK) overnight at 4°C. Protein A+G Agarose/Salmon Sperm DNA was used to precipitate complexes recognized by primary antibodies. We removed non-specific binding through continuous washing. Elution buffer and NaCl are used for elution and removal of crosslinks. After purification, DNA was amplified and detected through PCR reaction.

#### Luciferase reporter assay

We predicted the binding sites of STAT3 in the promoter of HSPG2. Promoter fragments of HSPG2 containing STAT3 binding sites were inserted into the reporter plasmid to construct the wild-type reporter plasmid. We used the mutant plasmid as the control and transfected the related oligonucleotide and biological reagent and luciferase reporter plasmid into the cells. The Luciferase Report Assay System (Promega, USA) was used to detect the activity of luciferase.

#### Hematoxylin-eosin (HE), masson staining and immunohistochemistry (IHC) staining

For HE staining, sections were dewaxed in xylene and rehydrated through graded alcohol. Nuclei were stained with hematoxylin, and cytoplasmic components were stained with eosin. Sections were dehydrated in ascending ethanol, cleared in xylene, and sealed with neutral resin for microscopic imaging. The dermal thickness was measured as the perpendicular distance from the epidermal-dermal junction to the dermal-hypodermal junction, and OLYVIA software was used for measurement. For Masson staining, dewaxing and rehydration procedures were identical to HE staining, sections were sequentially stained with: the nuclei with hematoxylin, cytoplasm with acid fuchsin, the collagen with aniline blue. Dehydration and mounting paralleled the HE protocols. For IHC staining, antigen retrieval was performed using citric acid buffer after dewaxing and rehydration. Endogenous peroxidase activity was blocked with 3% hydrogen peroxide and methanol. Sections were incubated overnight at 4°C with primary antibodies: antibodies against RARRES2 (Proteintech, USA), HSPG2 (Abcam, Cambridgeshire, UK) and α-SMA (Affinity Biosciences, UK). HRP-conjugated goat anti-rabbit IgG polymer (ZSGB-BIO, China) was applied for incubating the sample. Diaminobenzidine (DAB) chromogenic development was followed by hematoxylin staining, dehydration, sealing, and photography. ImageJ software was used to quantitatively analyze the results of Masson and IHC staining.

### Quantification and statistical analysis

For MR analyses, the causal estimates were derived using IVW, Weighted mode, MR-Egger and Weighted median method. The Wald ratio method was used for single SNP analyses. Sensitivity analyses included tests for heterogeneity (Cochran's Q test in MR-Egger and IVW) and pleiotropy (MR-Egger intercept). For experimental data, results are expressed as mean ± standard deviation (SD). Statistical analysis was performed using GraphPad Prism software (Prism 8) for generating charts, with partial figures created through the online platform at https://www.bioinformatics.com.cn. Differences between two groups were assessed using Student's t-test, while one-way ANOVA was used for three or more groups. Spearman correlation was used to assess gene-expression relationships. Specific statistical details can be found in the figure legends. Key overall analysis results are reported in the results section. In all analyses, a statistically significant difference was defined as P<0.05. Statistical significance is denoted by asterisks (∗), with the exact P-value thresholds defined in each legend (e.g., ∗p < 0.05, ∗∗p < 0.01, ∗p < 0.001).

## Supplemental information

Document S1. Figures S1–S3

Table S1. 5909 SNPs related to 2940 plasma proteins were included as instrumental variables (UKB-PPP)

Table S2. 5615 SNPs related to 4907 plasma proteins were included as instrumental variables (deCODE genetics)

Table S3. 1098 SNPs related to 2897 plasma proteins were included as instrumental variables (FinnGen)

Table S4. SMR and HEIDI analyses in the eQTL of RARRES2

Table S5. MR-PheWAS results

Table S6. Differential expression analysis in 7 keloid tissues and 6 adjacent normal

Table S7. The hub-genes in MEturquoise module

Table S8. 788 key genes in keloid

Table S9. 796 genes related to lipid metabolism

Table S10. 27 lipid metabolism-related genes that play an important role in keloid

Table S11. CytoHubba plugin scores each gene

Table S12. 11 hub-genes of the PPI network
