# Supplementary material for: Integrative multiomics analysis identifies RARRES2 as a regulator of keloid pathogenesis through STAT3/HSPG2 signaling axis
Source: iScience. 2026 Apr 20;29(5):115797. doi: 10.1016/j.isci.2026.115797 (PMC13187529; doi:10.1016/j.isci.2026.115797)
Supplement: Document S1. Figures S1–S3 [file mmc1.pdf]

iScience, Volume *29*

Supplemental information

Integrative multiomics analysis identifies RARRES2 as a regulator of keloid pathogenesis through STAT3/HSPG2 signaling axis

Wenkang Luan, Shujun Fan, Hanyi Jiang, Dongwen Jiang, Jinxiu Yang, and Leren He

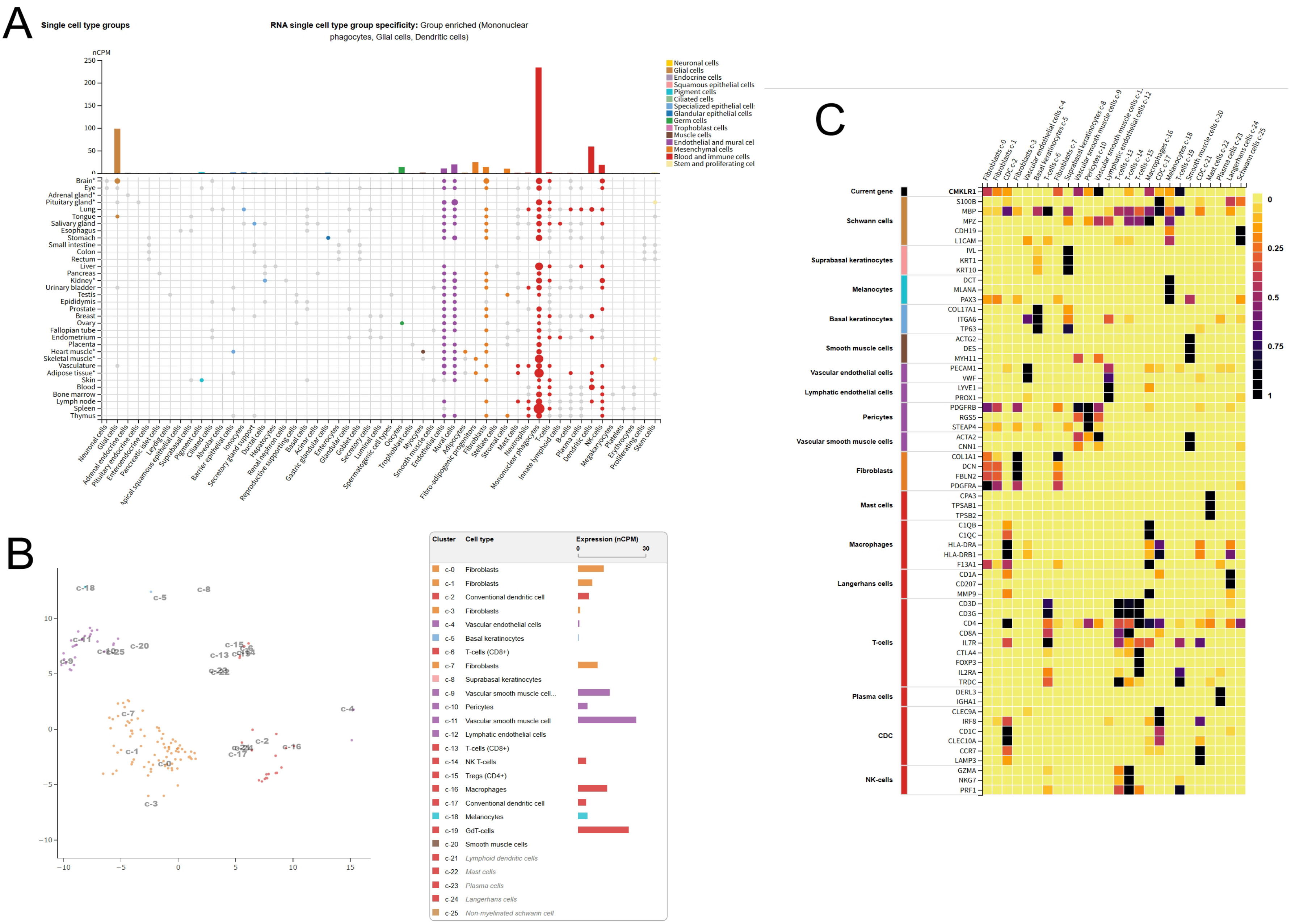

Supplementary Figure 1

(A) The mRNA expression of CMKLR1 in different cells of different tissues through Human Protein Atlas. (B) and (C) Single cell sequencing of skin tissues indicated that CMKLR1 is mainly enriched in in vascular smooth muscle cells and fibroblasts.

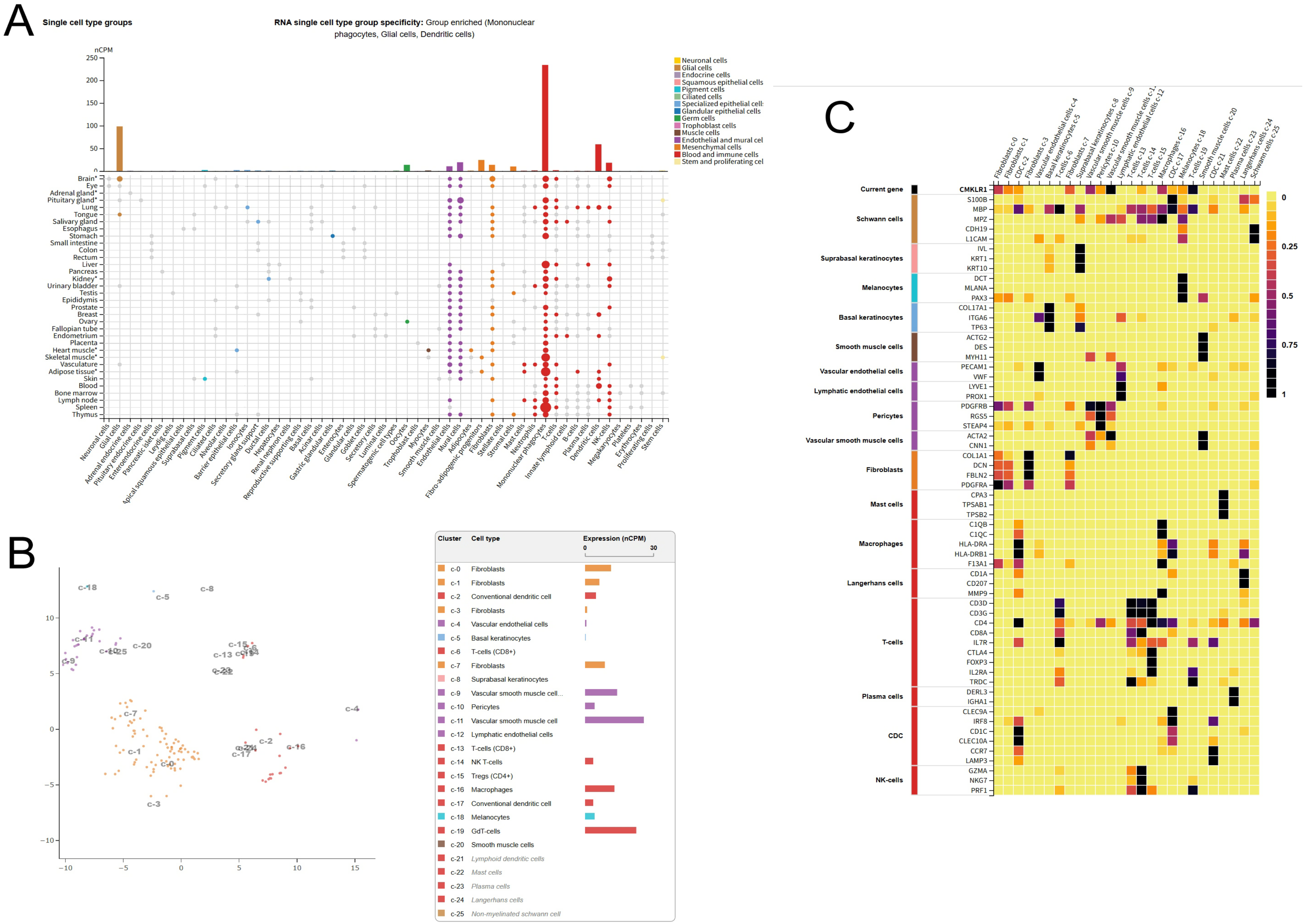

Supplementary Figure 2

(A) The morphology of primary fibroblasts. Scale bar, 100 μm. (B) Flow cytometry showed that primary fibroblasts mainly expressed the cell markers (CD90, FSP1 and vimentin).

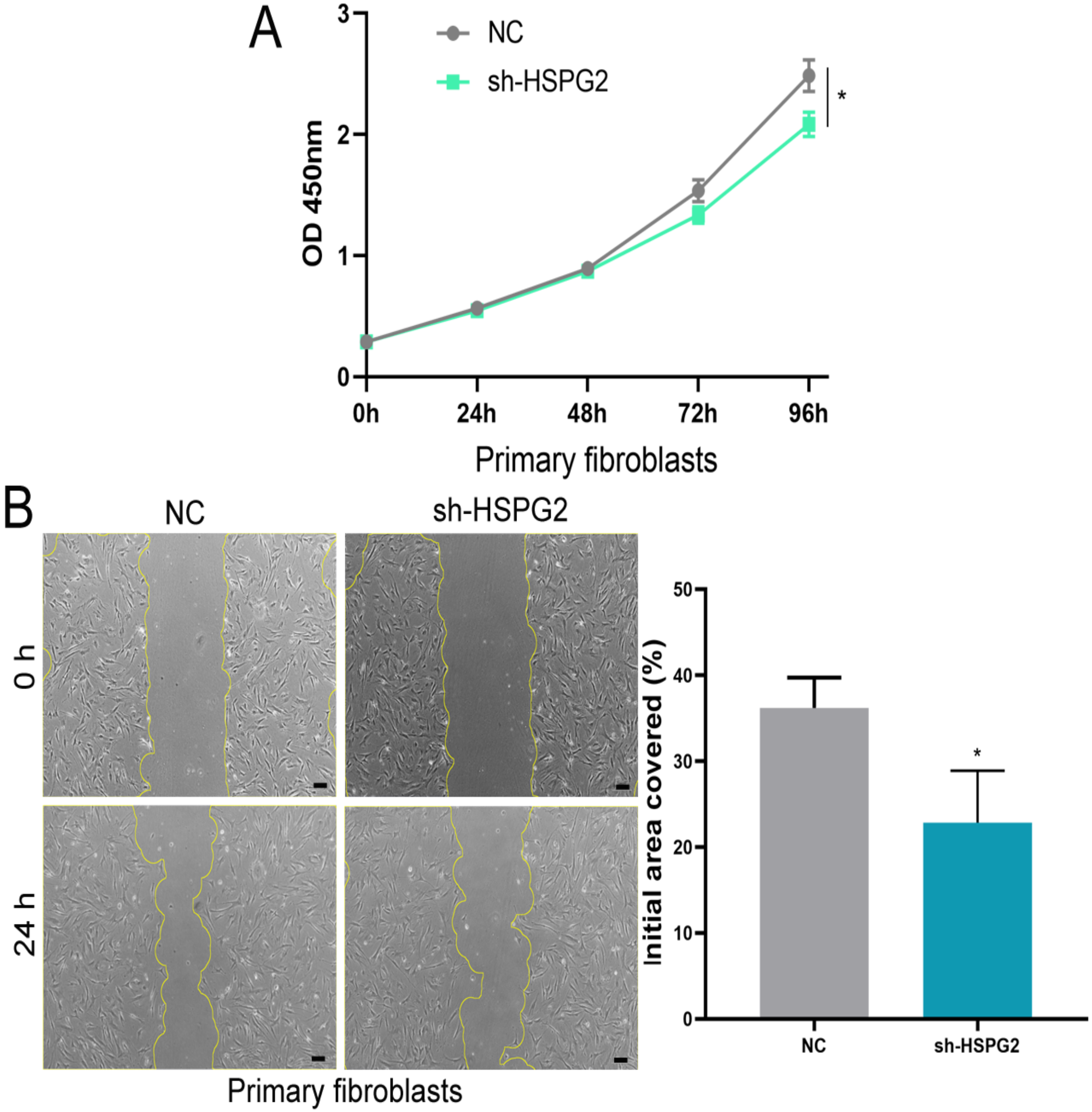

Supplementary Figure 3

(A) The proliferative ability of primary fibroblasts was determined by CCK8 assay in different groups. (B) The migration of primary fibroblasts in different groups was detected by scratch wound assay. n=3, Scale bar, 50 μm. *P < 0.05.
